# Urinary Modified C-Reactive Protein is Closely Associated with Tubulointerstitial Lesions in Lupus Nephritis

**DOI:** 10.1155/2023/6107911

**Published:** 2023-07-28

**Authors:** Mo Yuan, Xiao-ling Liu, Ying Tan, Feng Yu, Ming-hui Zhao

**Affiliations:** ^1^Renal Division, Peking University First Hospital, Beijing, China; ^2^Institute of Nephrology, Peking University, Beijing, China; ^3^Key Laboratory of Renal Disease, Ministry of Health of China, Beijing, China; ^4^Key Laboratory of CKD Prevention and Treatment, Ministry of Education of China, Beijing, China; ^5^Research Units of Diagnosis and Treatment of Immune-Mediated Kidney Diseases, Chinese Academy of Medical Sciences, Beijing, China; ^6^School of Medicine, Yunnan University, Kunming, China; ^7^Department of Pathology, Affiliated Hospital of Yunnan University, Second People's Hospital of Yunnan Province, Kunming, China; ^8^MOE Key Laboratory of Cell Activities and Stress Adaptations, School of Life Sciences, Lanzhou University, Lanzhou, China; ^9^Department of Nephrology, Peking University International Hospital, Beijing, China

## Abstract

**Objective:**

Modified C-reactive protein (mCRP) is known to be involved in the upregulation and amplification of the local inflammatory response. This study investigated the circulating and local levels of mCRP and their relevance to clinicopathological features in patients with lupus nephritis.

**Methods:**

Ninety-five patients with renal biopsy-proven lupus nephritis and 30 normal controls were enrolled in this study. Plasma and urinary mCRP were screened by enzyme-linked immunosorbent assay (ELISA). The renal deposition of mCRP was detected by immunohistochemistry and immunofluorescence staining. A human proximal tubular epithelial cell line (HK2 cells) was incubated with purified IgG from lupus nephritis, and the production of CRP by HK2 cells was further evaluated.

**Results:**

Plasma and urinary levels of mCRP increased significantly in patients with lupus nephritis compared with normal controls (*P* = 0.013, *P* < 0.001, respectively). The urinary mCRP levels were associated with interstitial inflammatory cell infiltration (*r* = 0.514, *P* < 0.001) and interstitial fibrosis (*r* = 0.270, *P* = 0.008). The ROC–AUC of the urinary mCRP levels for diagnosing tubulointerstitial lesions was 0.766. The urinary mCRP levels were closely associated with poor outcomes (HR: 1.204, 95% CI: 1.029–1.409, *P* = 0.020). However, no correlations were found of the plasma mCRP levels with clinicopathological data or the prognosis of lupus nephritis. CRP was mostly deposited in the renal tubules in patients with lupus nephritis, and the expression of CRP was significantly correlated with tubulointerstitial lesion indices. Immunofluorescence staining showed that mCRP could colocalize with IgG in tubules. Lupus nephritis-derived IgG could induce CRP production by HK2 cells.

**Conclusion:**

Urinary mCRP levels were significantly increased, and urinary mCRP might be a biomarker for tubulointerstitial lesions in patients with lupus nephritis. Renal CRP could be produced by tubular epithelial cells after stimulation by lupus nephritis-derived IgG, and the local presence of mCRP might play a critical role in the development of tubulointerstitial lesions.

## 1. Introduction

Systemic lupus erythematosus (SLE) is a prototypical multisystem autoimmune disease, and lupus nephritis is a common and severe manifestation of SLE that can result in irreversible renal impairment. In addition to glomerular injury, tubulointerstitial lesions could also be prominent in lupus nephritis [1]. Previous studies found that more than half of patients with lupus nephritis have tubulointerstitial abnormalities [2, 3]. Moreover, tubulointerstitial indices were significant independent risk factors for renal outcomes in lupus nephritis [4, 5]. The revision of the International Society of Nephrology/Renal Pathology Society (ISN/RPS) 2018 classification system for lupus nephritis further emphasized the importance of tubulointerstitial lesions [6]. However, there is a lack of reliable and appropriate biomarkers reflecting tubulointerstitial lesions in lupus nephritis patients.

Plasma native C-reactive protein (nCRP) has been widely recognized as a highly conserved acute phase reactant, primarily produced by hepatocytes in response to tissue injury or infection that elicits an inflammatory response [7, 8]. Thus, it is regarded as a nonspecific marker for ongoing inflammation in clinical practice. nCRP belongs to the pentraxin family, which consists of five identical nonglycosylated globular subunits [9]. When nCRP in the local inflammatory area encounters activated membranes [10–12], neutrophil extracellular traps [13], or acidic pH [14], biochemical forces contribute to dissociation of the pentamers into monomers. The dissociated conformation is termed modified CRP (mCRP), which exerts potent proinflammatory actions on endothelial cells, macrophages, and neutrophils and can upregulate and amplify the local inflammatory response [15–17].

Levels of nCRP can increase dramatically following an acute-phase stimulus. However, despite evident inflammation and tissue damage, SLE fails to elicit major nCRP production [18]. However, increased levels of plasma mCRP have been found in patients with skin-related inflammatory autoimmune disorders, such as eczema, psoriasis, and urticaria, whereas the levels of plasma nCRP in patients did not differ significantly from those in normal controls [19]. In addition, the expression of CRP mRNA and the synthesis of nCRP have been reported in renal tubular epithelial cells [20]. Schwedler et al. [21] detected tubular mCRP deposition in renal biopsies from patients with diabetic nephropathy, and local mCRP expression was correlated with the severity of histologically detectable lesions.

Thus, we investigated the circulating and local levels of mCRP in patients with lupus nephritis, and their correlations with clinicopathological features and prognosis were evaluated.

## 2. Materials and Methods

### 2.1. Patients and Samples

Complete clinicopathological data of 95 patients with renal biopsy-proven lupus nephritis diagnosed from January 2016 to July 2019 at Peking University First Hospital were collected for analysis. All patients fulfilled the 1997 American College of Rheumatology revised criteria for SLE [22]. The enrollment of patients with lupus nephritis is shown in Figure S1.

Plasma and morning urine samples from all of the patients were obtained on the day of renal biopsy before initiation of immunosuppressive treatment. Plasma and urine samples from 30 healthy volunteers, matched for age and sex, were selected as normal controls. The first plasma exchange samples from five patients with active lupus nephritis were collected upon presentation. Plasma samples from five healthy volunteers served as normal controls.

Renal biopsies of 20 patients with lupus nephritis from Peking University First Hospital were selected for the pathological study. Among these patients, four had severe tubulointerstitial nephritis, and another 16 had no (*n* = 5) or mild to moderate (*n* = 11) tubulointerstitial injury. Renal tissues from normal areas of nephrectomized kidneys from five patients with solitary renal cell carcinoma were collected as the normal controls.

All plasma and urine were stored in aliquots at −80°C until use, and repeated freeze–thaw cycles were avoided. Informed consent was obtained from each patient for blood and urine sampling and renal biopsy. The research was performed in accordance with the principles of the Declaration of Helsinki and was approved by the local ethical committees (number 2014 (749)).

### 2.2. Clinical Assessment

The following clinical data were recorded: sex, age at kidney biopsy, fever, eruption, photosensitivity, oral ulcer, alopecia, arthralgia, serositis, neurologic disorder, anemia, thrombocytopenia, leukocytopenia, hematuria, leukocyturia, and acute kidney injury (AKI) [23]. The Systemic Lupus Erythematosus Disease Activity Index (SLEDAI) was used to assess the clinical disease activity and progression of lupus nephritis patients [24, 25].

The patients were followed-up in outpatient lupus clinics. The primary endpoint was defined as death, and the secondary endpoints were defined as end-stage renal disease (ESRD), renal transplant, or doubling of serum creatinine levels.

### 2.3. Laboratory Assessment

An indirect immunofluorescence assay (EUROIMMUN, Lübeck, Germany) was used to detect serum antinuclear antibodies [26]. A *Crithidia luciliae* indirect immunofluorescence assay (EUROIMMUN, Lübeck, Germany) was used to detect anti-double-stranded DNA antibodies [26]. An enzyme-linked immunosorbent assay (ELISA) (EUROIMMUN, Lübeck, Germany) was used to detect anticardiolipin antibodies [26]. A rate nephelometry assay (IMMAGE; Beckman-Coulter, USA) was used to determine circulating C3 levels [26]. Urinary neutrophil gelatinase-associated lipocalin (NGAL) and kidney injury molecule-1 (KIM-1) were detected by commercial kits (R&D Systems, Minneapolis, MN, USA).

### 2.4. Renal Histopathology

The renal biopsy specimens were processed using light microscopy, direct immunofluorescence, and electron microscopy. Lupus nephritis classification was determined according to the ISN/RPS 2018 classification system [6]. Pathological parameters, including activity indices and chronicity indices, were assessed by two experienced nephropathologists using a previously reported system involving semiquantitative scoring of specific biopsy features [27, 28]. Activity indices (AIs) included endocapillary hypercellularity, neutrophils/karyorrhexis, fibrinoid necrosis, cellular-fibrocellular crescents, subendothelial hyaline deposits, and interstitial inflammation, whereas the chronicity indices (CIs) included glomerular sclerosis, fibrous crescents, tubular atrophy, and interstitial fibrosis. Tubulointerstitial lesions were semiquantitatively scored according to the affected area of the tubulointerstitium. The degree of tubulointerstitial lesions was graded as normal, mild (<25%), moderate (25%–50%), and severe (>50%), and scores of 0, 1, 2, and 3 were assigned, respectively [4].

### 2.5. Detection of Plasma and Urinary mCRP Levels Using ELISA

According to previous research [19], mouse monoclonal antihuman CRP antibodies (clone 8, Sigma–Aldrich, St. Louis, MO, USA) were coated onto microtiter wells (Thermo Fisher Scientific, Waltham, MA, USA) at 1 : 1,000 in coating buffer (10 mM sodium carbonate/bicarbonate, pH 9.6) overnight at 4°C. All the following steps were conducted at 37°C. The plates were blocked with 1% bovine serum albumin (BSA) in TBS (10 mM Tris, 140 mM NaCl, 2 mM Ca, pH 7.4) (blocking buffer) for 1 hr. Diluted samples in blocking buffer were added to the wells and incubated for 1 hr. Then, the sheep antihuman CRP polyclonal antibody (BindingSite, Birmingham, UK) diluted to 1 : 2,000 in blocking buffer was added to the wells and incubated for 1 hr. The plates were incubated with HRP-labeled donkey antisheep IgG (H + L) (Abcam, Cambridge, MA, USA) for 1 hr. After incubation, the wells were developed with a 3,3′, 5,5′-tetramethylbenzidine (TMB) and stopped with 1 M H_2_SO_4_. The results were measured with a microplate reader at 450 and 570 nm. Serial concentrations of urea-denatured mCRP standards from 0 to 20 ng/ml were used to develop a standard curve.

### 2.6. Detection of Renal Expression of CRP by Immunohistochemistry Assay

Renal tissue samples were fixed in 4% buffered paraformaldehyde and embedded in paraffin. Deparaffinized slides were heated in a pressure cooker with EDTA buffer (pH 9) for 5 min. After heat-mediated antigen retrieval, the slides were immersed in freshly prepared 3% hydrogen peroxide for 30 min at room temperature to quench endogenous peroxidase activity. To block nonspecific staining, the slides were incubated with 3% BSA in phosphate-buffered saline (PBS) at room temperature for 1 hr. The primary anti-CRP antibodies (Abcam), which are supposed to recognize nCRP and mCRP according to the product description, were directly added to each slide and incubated overnight at 4°C. The secondary antibodies and staining were performed using a commercial kit (ZGSB-BIO, Beijing, China). The negative control used PBS instead of primary antibody. Image-Pro Plus analysis, software 6.0 (Media Cybernetics, Dallas, TX, USA), was used to evaluate the mean optical density of CRP staining of renal tubules.

### 2.7. Colocalization of mCRP and IgG in the Kidney by Immunofluorescence Staining

Fresh-frozen sections were fixed in cold acetone for 20 min, followed by blocking with 3% BSA for 1 hr at room temperature. The sections were incubated with primary anti-CRP antibodies (Sigma) overnight at 4°C. The blank control used PBS instead of primary antibody. After extensive washing, rhodamine (TRITC)-conjugated goat antimouse IgG (H + L) (Sigma) was used as a secondary antibody for 1 hr at 37°C. Then, the sections were stained with Alexa Fluor 488-conjugated goat antihuman IgG (H + L) (Jackson Immunoresearch Laboratories, West Grove, PA, USA) for 1 hr at 37°C. After DAPI staining, the sections were examined using a confocal microscope (Olympus Viewer 1000, Tokyo, Japan).

### 2.8. Purification of IgG

IgG was purified from the plasma exchange of five lupus nephritis patients and the plasma of five normal individuals by a protein G affinity column on an AKTA–FPLC system (GE Biosciences, South San Francisco, CA, USA) [29].

### 2.9. Analysis of the Levels of nCRP and mCRP in the Cell Supernatant by ELISA

The normal human proximal tubular epithelial cell line (HK2 cells) from American Type Culture Collection (ATCC, Manassas, VA, USA) was cultured in DMEM-F12 medium (Gibco, Grand Island, NY, USA) supplemented with 10% fetal bovine serum (Gibco) and 1% penicillin/streptomycin antibiotics (Gibco) at 37°C and 5% CO_2_.

After incubation of HK2 cells with purified IgG (100 *μ*g/ml) for 24–72 hr, the supernatant was collected and centrifuged at 2000 × *g* for 10 min to remove cell debris, and the levels of nCRP and mCRP were measured by homemade ELISA according to a previous study [19].

### 2.10. RNA Extraction and Polymerase Chain Reaction (PCR)

After incubation of HK2 cells with purified IgG for 24–72 hr, total RNA was extracted from the HK2 cells using the RNeasy Mini Kit (TIANGEN Biotech, Beijing, China). Complementary DNA (cDNA) was synthesized from total RNA (1 *μ*g) using high-capacity cDNA reverse transcription kits (Vazyme Biotech, Nanjing, China) in accordance with the method provided by the manufacturer. Quantitative real-time PCR was performed using SYBR Green Master Mix reagent (Thermo Fisher Scientific). The housekeeping gene GAPDH served as an internal control. The human CRP primer sequences were: forward 5′-GTCACAGTAGCTCCAGTACACA-3′; and reverse 5′-AAAGTTCCCACCGAAGGAATC-3′. The ratio of CRP mRNA to GAPDH mRNA was analyzed using the 2^^−*ΔΔ*Ct^ method.

### 2.11. Statistical Analysis

SPSS software, version 26.0 (SPSS Inc., Chicago, IL, USA), was applied for statistical analysis. Continuous variables are expressed as the mean ± standard deviation (SD) or median (interquartile range (IQR)), and categorical variables are expressed as ratios. The associations between continuous variables were performed using Pearson's correlation analysis or Spearman's rank correlation analysis. Statistical analysis was performed with the independent-sample *t*-test, the Mann–Whitney *U* test, one-way ANOVA or the Kruskal–Wallis test as appropriate. Receiver-operating characteristic (ROC) curves were constructed, and the area under the curve (AUC) was used to distinguish tubulointerstitial lesions. Kaplan–Meier curves were employed to test patient prognosis. Univariate survival analysis was performed using the log rank test. A two-sided *P* < 0.05 was defined as statistically significant.

## 3. Results

### 3.1. General Data of Patients with Lupus Nephritis

The detailed general data of patients with lupus nephritis are shown in Table S1. Twenty-two (23.2%) were male, and 73 (76.8%) were female, with a mean age of 32.5 ± 13.0 years old. According to the ISN/RPS classification system of lupus nephritis, two patients were classified as class II (2.1%), 27 as class III (28.4%, including 11 with class V + III), 51 as class IV (53.7%, including 16 with class V + IV), and 15 as class V (15.8%).

### 3.2. Plasma mCRP Levels and Urinary mCRP Levels in Patients with Lupus Nephritis and Controls

**Figure 1:**
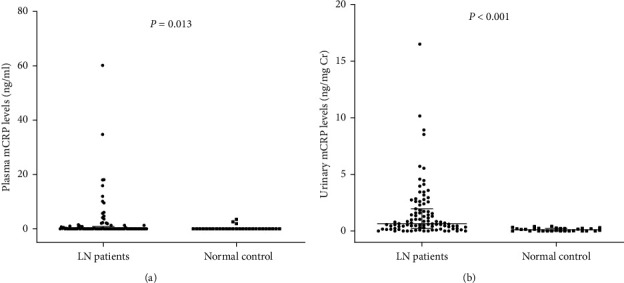
Plasma mCRP levels and urinary mCRP levels in different groups: (a) plasma mCRP levels in patients with lupus nephritis and normal controls; (b) urinary mCRP levels in patients with lupus nephritis and normal controls. mCRP, modified C reactive protein; LN, lupus nephritis.

The plasma level of mCRP in lupus nephritis patients was significantly higher than that in normal controls (0 (0–0.85) ng/ml versus 0 (0–0) ng/ml, *P* = 0.013). In addition, the urinary level of mCRP in lupus nephritis patients was significantly higher than that in normal controls (0.66 (0.23–1.96) ng/mg Cr vs. 0.03 (0–0.24) ng/mg Cr, *P* < 0.001) (Figure 1).

### 3.3. Associations between Plasma mCRP Levels or Urinary mCRP Levels and Clinicopathological Features of Lupus Nephritis

No correlations were found between the plasma mCRP levels and clinicopathological data of lupus nephritis patients (Table 1). Moreover, there were no associations between plasma mCRP levels and urinary NGAL levels (*r* = 0.063, *P* = 0.545) or urinary KIM-1 levels (*r* = 0.063, *P* = 0.549) in patients with lupus nephritis (Figure S2 A1, B1).

Further analysis indicated that, in the patients with lupus nephritis, the levels of urinary mCRP were significantly correlated with serum creatinine (*r* = 0.460, *P* < 0.001), urinary NGAL levels (*r* = 0.390, *P* < 0.001) and urinary KIM-1 levels (*r* = 0.227, *P* = 0.028) (Figure S2 A2, B2). With regard to renal pathological parameters, the levels of urinary mCRP were positively correlated with activity index scores (*r* = 0.214, *P* = 0.038), endocapillary hypercellularity (*r* = 0.216, *P* = 0.036), interstitial inflammatory cell infiltration (*r* = 0.514, *P* < 0.001), chronicity index scores (*r* = 0.292, *P* = 0.004), glomerular sclerosis (*r* = 0.241, *P* = 0.019), and interstitial fibrosis (*r* = 0.270, *P* = 0.008) in patients with lupus nephritis (Table 1).

### 3.4. Urinary mCRP Levels Can Distinguish Tubulointerstitial Lesions

**Figure 2:**
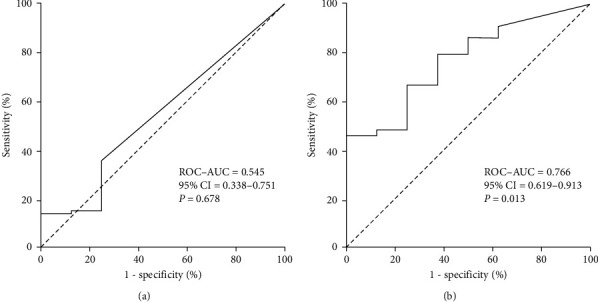
The predictive value of plasma mCRP levels and urinary mCRP levels for tubulointerstitial injury: (a) ROC curves for predicting tubulointerstitial lesions based on the plasma mCRP levels; (b) ROC curves for predicting tubulointerstitial lesions based on the urinary mCRP levels. ROC-AUC, receiver operating characteristic-area under the curve; 95% CI, 95% confidence interval.

For distinguishing tubulointerstitial lesions in patients with lupus nephritis, the ROC–AUC of the plasma mCRP levels was 0.545 (95% confidence interval (CI): 0.338–0.751, *P* = 0.678) (Figure 2(a)). The urinary mCRP levels augmented the ROC–AUC to 0.766 for predicting tubulointerstitial lesions (95% CI: 0.619–0.913, *P* = 0.013) (Figure 2(b)). An assay of urinary mCRP levels could identify tubulointerstitial lesions with sensitivity of 79.3% and specificity of 62.5%.

### 3.5. Associations of Plasma mCRP Levels or Urinary mCRP Levels and Composite Outcomes in Patients with Lupus Nephritis

**Figure 3:**
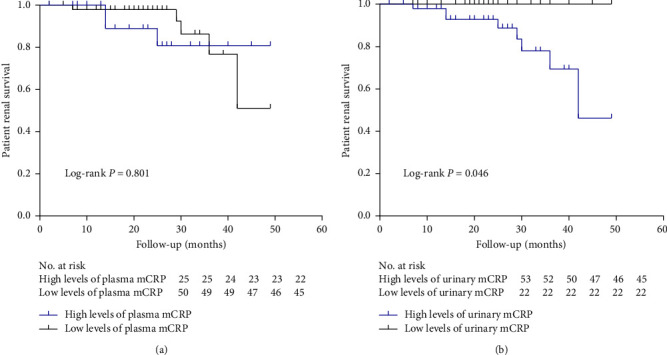
Kaplan–Meier analysis of composite endpoints between the high levels of mCRP group and the low levels of mCRP group: (a) Kaplan–Meier analysis of composite endpoints between the high levels of plasma mCRP group and the low levels of plasma mCRP group in lupus nephritis; (b) Kaplan–Meier analysis of composite endpoints between the high levels of urinary mCRP group and the low levels of urinary mCRP group in lupus nephritis. mCRP, modified C reactive protein.

In our research, 75 patients with lupus nephritis were followed-up regularly, and the average follow-up time was 22.8 ± 11.7 months. In terms of long-term outcomes, one patient died (1/75%, 1.3%), five patients reached ESRD (5/75%, 6.7%), one patient doubled in serum creatinine levels (1/75%, 1.3%), and one patient received a kidney transplant (1/75%, 1.3%). Importantly, the Kaplan–Meier curve demonstrated that the patients with the higher levels of urinary mCRP (>0.391 ng/ml, the mean value of urinary mCRP plus two times the SD in normal controls) had significantly worse prognoses (*P* = 0.046) (Figure 3(b)). A subsequent univariate survival analysis revealed that the levels of urinary mCRP were a risk factor for prognosis (hazard ratio (HR): 1.204, 95% CI: 1.029–1.409, *P* = 0.020) (Table 2). In contrast, the levels of plasma mCRP were not associated with the prognosis of lupus nephritis (HR: 0.979, 95% CI: 0.798–1.201, *P* = 0.836) (Figure 3(a)) (Table 2).

### 3.6. Expression of CRP in Renal Tissues in Lupus Nephritis Patients and Its Pathological Associations

**Figure 4:**
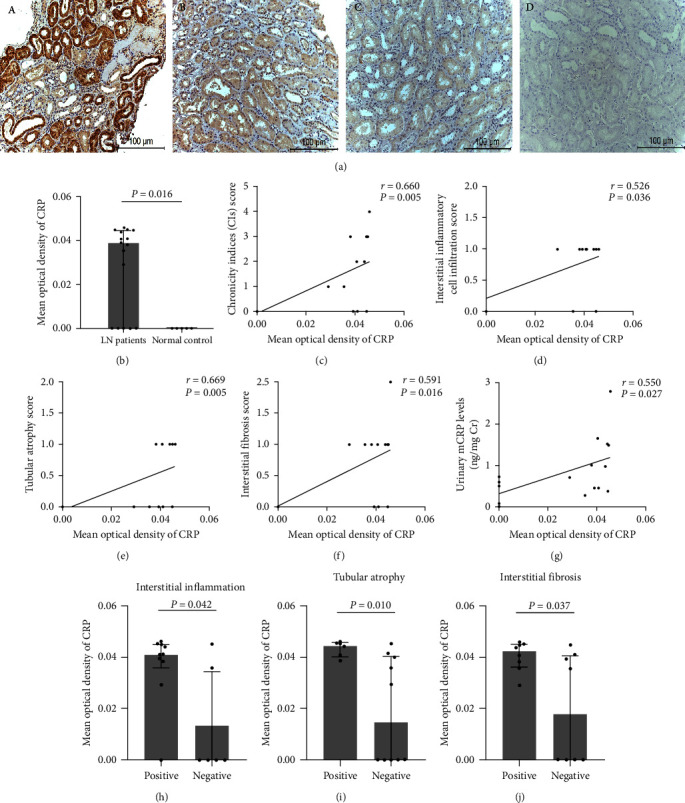
Immunohistochemistry staining of CRP in renal biopsies from patients with lupus nephritis and normal controls: (a(A)) CRP staining was positive in the tubules of patients with lupus nephritis (arrow). (a(B)) CRP staining was negative in patients with lupus nephritis. (a(C)) CRP staining was barely seen in the tubules of normal kidneys. (a(D)) Blank control (×400). Scale bars 100 *μ*m; (b) the mean optical density of CRP in patients with lupus nephritis and normal controls; (c–f) the associations between the expression of CRP and pathologic indices; (g) associations between the expression of CRP and the levels of urinary mCRP; (h–j) comparisons of the expression of CRP between patients with tubulointerstitial lesions and patients without tubulointerstitial lesions. CRP, C reactive protein; LN, lupus nephritis.

CRP staining was observed in the cytoplasm of tubules in 11 of 16 renal biopsies from patients with lupus nephritis (class III: 3; class IV: 6; and class V: 7) (Figure 4(a)). The expression of CRP in lupus nephritis patients was significantly higher than that in the normal controls (*P* = 0.016) (Figure 4(b)). In addition, CRP could be detected in the tubules of four patients with lupus-related tubulointerstitial nephritis, and the mean optical density of CRP in these patients was significantly higher than that in the normal controls (*P* = 0.007) (Figure S3).

Furthermore, the mean optical density of CRP was positively correlated with chronicity index scores (*r* = 0.660, *P* = 0.005), interstitial inflammatory cell infiltration (*r* = 0.526, *P* = 0.036), tubular atrophy (*r* = 0.669, *P* = 0.005), and interstitial fibrosis (*r* = 0.591, *P* = 0.016) (Figure 4(c)–4(f)). In particular, positive interstitial inflammation, tubular atrophy, and interstitial fibrosis corresponded to higher expression of CRP (*P* = 0.042; *P* = 0.010; *P* = 0.037, respectively) (Figure 4(h)–4(j)). In addition, there were significantly positive correlations between the expression of CRP in tubules and the levels of urinary mCRP in patients with lupus nephritis (*r* = 0.550, *P* = 0.027) (Figure 4(g)).

### 3.7. Co-localization of mCRP and IgG in Patients with Lupus Nephritis

**Figure 5:**
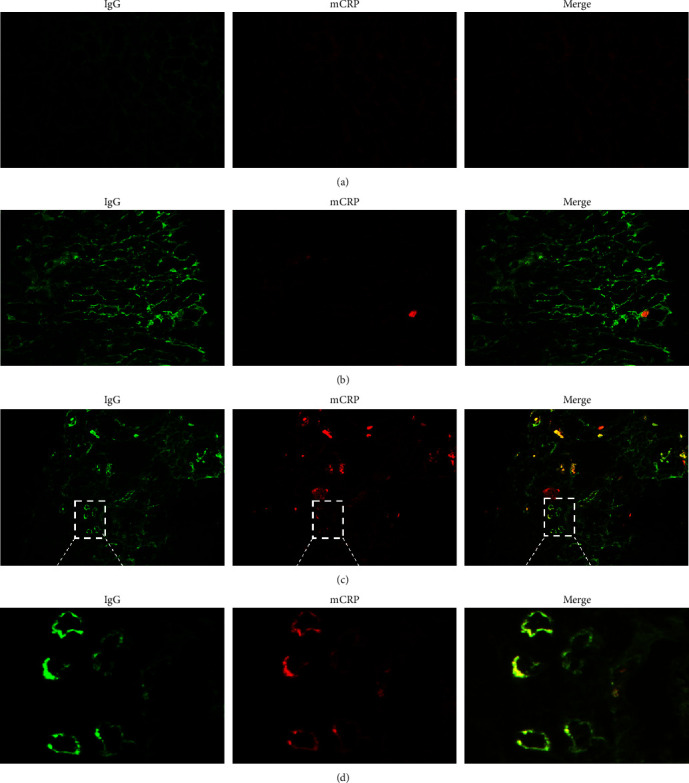
Colocalization of mCRP and IgG from patients with lupus nephritis: (a) the staining of mCRP and IgG was negative in the tubules of samples from normal controls; (b) blank control (PBS instead of primary anti-CRP antibodies); (c–d) mCRP was expressed in tubules of samples from patients with lupus nephritis, and IgG was deposited in the same areas. The renal staining of mCRP and IgG was partially merged. mCRP, modified C reactive protein.

Double immunofluorescence assays showed that mCRP was expressed in tubules of the kidneys obtained from patients with lupus nephritis, and IgG was deposited in the same areas. The renal staining of mCRP and IgG was partially merged. The staining of mCRP was scarcely seen in the tubules of samples from normal controls (Figure 5).

### 3.8. CRP Production and Secretion by HK2 Cells In Vitro

**Figure 6:**
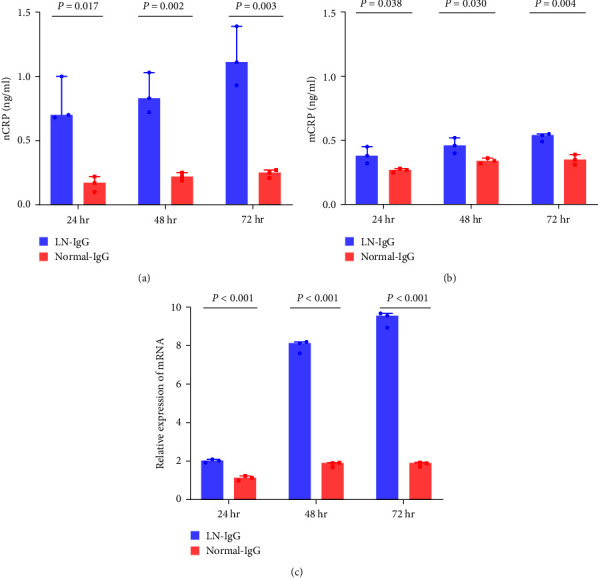
CRP production and secretion by HK2 cells: (a) the levels of nCRP; (b) mCRP in the supernatant of HK2 cells treated with IgG from patients with lupus nephritis for 24–72 hr were significantly higher than those exposed to normal IgG; (c) the expression of CRP mRNA in HK2 cells stimulated with IgG from patients with lupus nephritis for 24–72 hr was significantly higher than that in HK2 cells exposed to normal IgG. The above experiments were repeated three times. nCRP, native C reactive protein; mCRP, modified C reactive protein; LN, lupus nephritis.

To explore the ability of renal tubular epithelial cells to synthesize CRP, we incubated HK2 cells with purified IgG for 24–72 hr. As shown in Figures 6(a) and6(b), the levels of both nCRP and mCRP in the supernatant of HK2 cells treated with IgG from patients with lupus nephritis for 24–72 hr were significantly higher than those exposed to normal IgG. The above results were also verified by quantitative real-time PCR (Figure 6(c)).

## 4. Discussion

Tubulointerstitial lesions are frequently observed in lupus nephritis, and increasing evidence has shown that tubulointerstitial lesions are a potent predictor of poor outcome [3, 4]. Thus, the importance of tubulointerstitial lesions in lupus nephritis has been widely recognized. However, the pathogenesis of tubulointerstitial lesions in lupus nephritis is not entirely clear, and there is a lack of reliable and appropriate biomarkers reflecting tubulointerstitial damage in patients with lupus nephritis.

Our study identified for the first time that the levels of mCRP in plasma and urine increased significantly in lupus nephritis patients compared with normal controls. Further analysis showed that urinary mCRP levels were significantly correlated with urinary NGAL and KIM-1 levels, which are sensitive indicators of tubulointerstitial injury in lupus nephritis [30]. Regarding renal histopathology evaluation, urinary mCRP levels were also correlated with some tubulointerstitial lesion indices, of which interstitial inflammatory cell infiltration was the strongest. However, no correlations were found between the plasma mCRP levels and the clinicopathological data of lupus nephritis. Thus, our results indicated that urinary mCRP levels, rather than circulating mCRP levels, might be a biomarker of tubulointerstitial injury in lupus nephritis. Furthermore, urinary mCRP levels were valuable for predicting tubulointerstitial lesions (ROC–AUC > 0.700) in our study. More importantly, it was found that the levels of plasma mCRP were not associated with the prognosis of lupus nephritis, whereas urinary mCRP levels were closely associated with poor outcomes according to the univariate survival analysis. These findings supported that urinary mCRP levels could be a useful biomarker in predicting prognosis in lupus nephritis. Notably, our hospital is a tertiary referral center, and some patients were already in very critical condition at the time of admission; thus, the incidence of endpoint events could have been higher.

Based on the above research, we raised the possibility that renal locally produced mCRP might be a major source of urinary mCRP in lupus nephritis. To verify our hypothesis, we detected CRP expression in renal biopsies from lupus nephritis patients using an immunohistochemistry assay. Nonspecific staining of renal tissues appeared under the conditions of using the anti-mCRP-3H12 antibodies (residues 199–206) [31], provided by the cooperative laboratory, or the commercial monoclonal anti-CRP antibody clone 8, so the primary anti-CRP antibodies from Abcam, which are supposed to recognize both nCRP and mCRP, were chosen. Our staining results showed that CRP was mainly detected in the tubule areas of lupus nephritis patients and patients with autoimmune-related tubulointerstitial nephritis, but was negative in the glomeruli. The mean optical density of CRP was significantly correlated with tubulointerstitial lesion indices, such as interstitial inflammatory cell infiltration, tubular atrophy, and interstitial fibrosis in lupus nephritis. These results supported the strong expression of CRP in renal tissues during tubulointerstitial injury. A previous study showed that tubular CRP staining was increased with declining renal function and increasing severity of histological lesions in patients with advanced diabetic nephropathy [21]. In addition, our research showed that CRP staining in tubules was significantly correlated with urinary mCRP levels in patients with lupus nephritis. Thus, the renal expression of mCRP might be a major source of urinary mCRP in lupus nephritis.

Moreover, double staining by immunofluorescence revealed that mCRP was indeed deposited in tubules, and the colocalization of mCRP and IgG in patients with lupus nephritis could be detected. Previous studies, including ours, indicated that the circulating level of autoantibodies against mCRP is prevalent in patients with lupus nephritis and has been associated with tubulointerstitial lesions [32, 33]. A previous study reported that antidouble stranded DNA antibodies can bind to tubular epithelial cells, stimulate the secretion of the proinflammatory cytokines IL-6, interleukin 1*β* (IL-1*β*), and tumor necrosis factor *α* (TNF-*α*) [34] and promote the recruitment of inflammatory cells and the development of interstitial inflammation. In addition, Kinloch et al. [35] found that vimentin was highly expressed by tubulointerstitial inflammatory cells and that antivimentin antibodies might be involved in the in situ adaptive immune mechanism of tubulointerstitial inflammation. Thus, we speculated that the local presence of mCRP antigen not only was a biomarker for tubulointerstitial injury, but also participated in the pathogenesis of lupus nephritis. Anti-mCRP autoantibodies and mCRP might form immune complexes in situ in the tubulointerstitium, resulting in tubule injury, interstitial inflammation, and fibrosis, but the exact role of mCRP in the pathogenesis of lupus nephritis must be further explored.

More importantly, under exposure to lupus nephritis-derived IgG, increased nCRP and mCRP levels were detected in the supernatant of tubular epithelial cells. Previous studies have indicated that lupus nephritis was associated with disturbed apoptosis and impaired clearance, which might lead to the disruption of tolerance against autoantigens and the generation of autoantibodies [36, 37]. Our results showed that these autoantibodies could also induce CRP antigen expression by tubular epithelial cells and might be involved in the progression of lupus nephritis.

However, our study had some limitations. First, whether mCRP is dissociated from nCRP produced by renal tubular epithelial cells or directly synthesized in situ remains to be further studied. Second, the exact role of mCRP in the pathogenesis of tubulointerstitial injury in lupus nephritis requires further exploration.

In conclusion, our work showed that urinary levels of mCRP were significantly increased and that urinary mCRP might be a biomarker for tubulointerstitial lesions in patients with lupus nephritis. In addition, renal CRP could be produced by tubular epithelial cells, and the local presence of mCRP might play a critical role in the development of tubulointerstitial lesions.

## Figures and Tables

**Table 1:** Correlation analysis of plasma mCRP levels and urinary mCRP levels with clinicopathological data in lupus nephritis.

Clinicopathological data	Plasma mCRP levels		Urinary mCRP levels	
	*r* value (95% CI)	*P* value	*r* value (95% CI)	*P* value
Clinical data				
Age (years)	−0.087 (−0.289 to 0.120)	0.403	0.155 (−0.031 to 0.339)	0.133
SLEDAI	0.202 (−0.002 to 0.392)	0.050	0.138 (−0.070 to 0.326)	0.183
Hemoglobin (g/l)	−0.178 (−0.359 to 0.025)	0.085	−0.066 (−0.276 to 0.152)	0.524
Urine protein (g/24 hr)	0.073 (−0.117 to 0.276)	0.480	0.132 (−0.062 to 0.311)	0.202
Serum creatinine (*μ*mol/l)	0.194 (−0.006 to 0.378)	0.060	**0.460 (0.292 to 0.604)**	**<0.001**
Serum C3 (g/l)	0.005 (−0.208 to 0.214)	0.959	−0.062 (−0.266 to 0.144)	0.554
Serum C4 (g/l)	0.064 (−0.134 to 0.275)	0.540	0.051 (−0.144 to 0.253)	0.622
Renal histopathology indices				
Endocapillary hypercellularity	−0.005 (−0.192 to 0.188)	0.964	**0.216 (0.031 to 0.405)**	**0.036**
Neutrophils/karyorrhexis	−0.072 (−0.275 to 0.122)	0.490	0.092 (−0.093 to 0.268)	0.377
Fibrinoid necrosis	0.152 (−0.044 to 0.357)	0.142	−0.025 (−0.247 to 0.167)	0.808
Cellular–fibrocellular crescents	0.063 (−0.056 to 0.304)	0.545	0.040 (−0.178 to 0.241)	0.698
Subendothelial hyaline deposits	−0.022 (−0.201 to 0.161)	0.833	0.131 (−0.072 to 0.342)	0.204
Interstitial inflammatory cell infiltration	0.014 (−0.206 to 0.231)	0.895	**0.514 (0.363 to 0.655)**	**<0.001**
Activity indices score	0.110 (−0.082 to 0.315)	0.289	**0.214 (0.009 to 0.420)**	**0.038**
Glomerular sclerosis	−0.115 (−0.288 to 0.079)	0.265	**0.241 (0.041 to 0.431)**	**0.019**
Fibrous crescents	−0.002 (−0.160 to 0.195)	0.984	0.159 (−0.023 to 0.313)	0.123
Tubular atrophy	−0.035 (−0.241 to 0.159)	0.738	0.164 (−0.051 to 0.352)	0.113
Interstitial fibrosis	0.036 (−0.146 to 0.204)	0.730	**0.270 (0.065 to 0.460)**	**0.008**
Chronicity indices score	−0.047 (−0.242 to 0.153)	0.653	**0.292 (0.089 to 0.459)**	**0.004**

mCRP, modified C reactive protein; SLEDAI, Systemic Lupus Erythematosus Disease Activity Index; 95% CI, 95% confidence interval. Bold values are statistically significant.

**Table 2:** Univariate analysis of composite outcomes for patients with lupus nephritis.

Clinicopathological data	HR	95% CI	*P* value
Age	1.041	(0.987–1.098)	0.142
Sex	0.191	(0.047–0.773)	**0.020**
SLEDAI	0.992	(0.866–1.137)	0.914
Proteinuria	1.084	(0.986–1.192)	0.096
Hematuria	1.076	(0.129–8.983)	0.946
Leukocyturia	0.905	(0.225–3.646)	0.888
Acute kidney injury	4.544	(1.006–20.525)	**0.049**
Serum creatinine	1.010	(1.005–1.015)	**<0.001**
Plasma C3 level	3.273	(0.778–13.767)	0.106
Activity indices score	1.268	(1.014–1.585)	**0.037**
Chronicity indices score	1.396	(1.091–1.788)	**0.008**
Plasma mCRP levels	0.979	(0.798–1.201)	0.836
Urinary mCRP levels	1.204	(1.029–1.409)	**0.020**

HR, hazard ratio; 95% CI, 95% confidence interval; SLEDAI, Systemic Lupus Erythematosus Disease Activity Index; mCRP, modified C reactive protein. Bold values are statistically significant.

## Data Availability

The data that support the findings of this study are available from the corresponding author upon reasonable request.

## Supplementary Materials

Supplementary Materials Table S1: General data of patients with lupus nephritis. Figure S1: Enrollment of patients with lupus nephritis at Peking University First Hospital. HBV, hepatitis B virus. Figure S2: Associations between plasma mCRP levels or urinary mCRP levels and tubulointerstitial injury features of lupus nephritis. (A1, B1) The association between plasma mCRP levels and urinary NGAL levels and KIM-1 levels in patients with lupus nephritis. (A2, B2) The associations between urinary mCRP levels and urinary NGAL levels and KIM-1 levels in patients with lupus nephritis. mCRP, modified C reactive protein, NGAL, neutrophil gelatinase-associated lipocalin; KIM-1, kidney injury molecule-1. Figure S3: Immunohistochemistry staining of CRP in renal biopsies of patients with autoimmune-related tubulointerstitial nephritis. (a(A)) CRP staining was markedly positive in the tubules of patients with lupus-related tubulointerstitial nephritis (arrow). (a(B)) CRP staining was barely seen in the tubules of normal kidneys (×400). Scale bars 100 *μ*m. (b) The mean optical density of CRP in patients with lupus-related tubulointerstitial nephritis and normal controls. CRP, C reactive protein; LN, lupus nephritis.
