# Supplementary material for: Urinary Modified C-Reactive Protein is Closely Associated with Tubulointerstitial Lesions in Lupus Nephritis
Source: Mediators Inflamm. 2023 Jul 28;2023:6107911. doi: 10.1155/2023/6107911 (PMC10403327; doi:10.1155/2023/6107911)
Supplement: Supplementary Materials — Table S1: General data of patients with lupus nephritis. Figure S1: Enrollment of patients with lupus nephritis at Peking University First Hospital. HBV, hepatitis B virus. Figure S2: Associations between plasma mCRP levels or urinary mCRP levels and tubulointerstitial injury features of lupus nephritis. (A1, B1) The association between plasma mCRP levels and urinary NGAL levels and KIM-1 levels in patients with lupus nephritis. (A2, B2) The associations between urinary mCRP levels and urinary NGAL levels and KIM-1 levels in patients with lupus nephritis. mCRP, modified C reactive protein, NGAL, neutrophil gelatinase-associated lipocalin; KIM-1, kidney injury molecule-1. Figure S3: Immunohistochemistry staining of CRP in renal biopsies of patients with autoimmune-related tubulointerstitial nephritis. (a(A)) CRP staining was markedly positive in the tubules of patients with lupus-related tubulointerstitial nephritis (arrow). (a(B)) CRP staining was barely seen in the tubules of normal kidneys (×400). Scale bars 100 μm. (b) The mean optical density of CRP in patients with lupus-related tubulointerstitial nephritis and normal controls. CRP, C reactive protein; LN, lupus nephritis. [file 6107911.f1.docx]

**TABLE S1:** General data of patients with lupus nephritis.

| ***Clinical evaluation*** |  |
| --- | --- |
| Sex (male/female) | 22/73 |
| Age (mean ± SD) (years) | 32.5±13.0 |
| SLEDAI (median, IQR) | 19;14-21 |
| Anemia no. (%) | 80(84.2%) |
| Thrombocytopenia no. (%) | 23(24.2%) |
| Leukocytopenia no. (%) | 12(12.6%) |
| Hematuria no. (%) | 80(84.2%) |
| Leukocyturia (noninfection) no. (%) | 59(62.1%) |
| Acute kidney injury no. (%) | 32(33.7%) |
| ***Laboratory assessment*** |  |
| Hemoglobin (mean ± SD) (g/l) | 98.4±20.3 |
| Urine protein (median, IQR) (g/24 h) | 3.7;2.2-6.7 |
| Serum creatinine (median, IQR) (umol/l) | 114.6;73.9-184.5 |
| Antinuclear antibody (+) no. (%) | 94(98.9%) |
| Anti-dsDNA antibodies (+) no. (%) | 80(84.2%) |
| Anti-cardiolipin antibodies (+) no. (%) | 9(9.5%) |
| Serum C3 (median, IQR) (g/l) | 0.4;0.3-0.5 |
| ***Renal histopathology indices*** |  |
| Endocapillary hypercellularity (median, IQR) | 2;1-3 |
| Neutrophils/karyorrhexis (median, IQR) | 1;0-1 |
| Fibrinoid necrosis (median, IQR) | 0;0-2 |
| Cellular-fibrocellular crescents (median, IQR) | 2;0-4 |
| Subendothelial hyaline deposits (median, IQR) | 0;0-1 |
| Interstitial inflammatory cell infiltration (median, IQR) | 1;1-2 |
| Activity indices score (median, IQR) | 8;5-11 |
| Glomerular sclerosis (median, IQR) | 0;0-1 |
| Fibrous crescents (median, IQR) | 0;0-0 |
| Tubular atrophy (median, IQR) | 1;0-1 |
| Interstitial fibrosis (median, IQR) | 1;0-1 |
| Chronicity indices score (median, IQR) | 2;0-3 |

SD: standard deviation; IQR: interquartile range; SLEDAI: Systemic Lupus Erythematosus Disease Activity Index; dsDNA: double-stranded DNA.


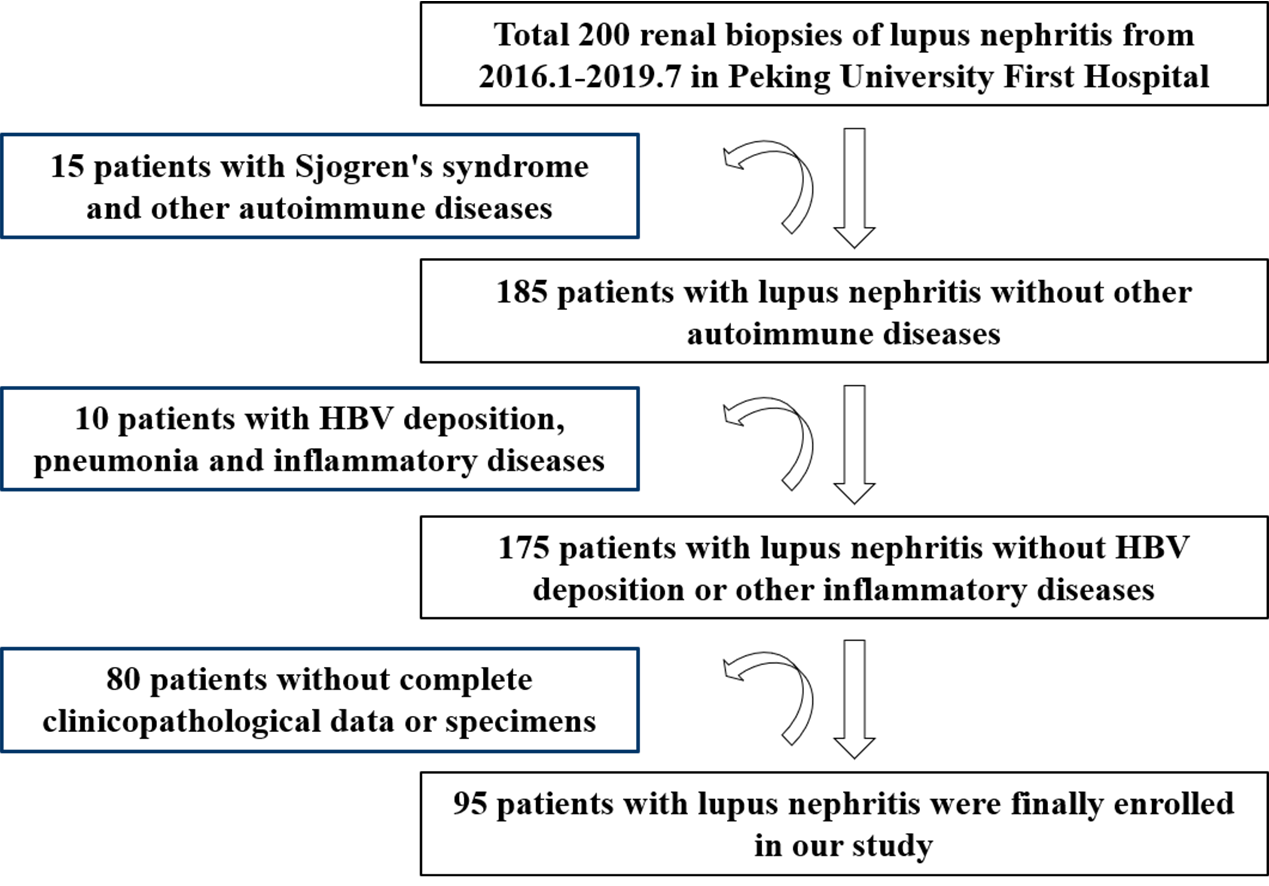


**Supplementary Figure S1:** Enrollment of patients with lupus nephritis at Peking University First Hospital. HBV: hepatitis B virus.


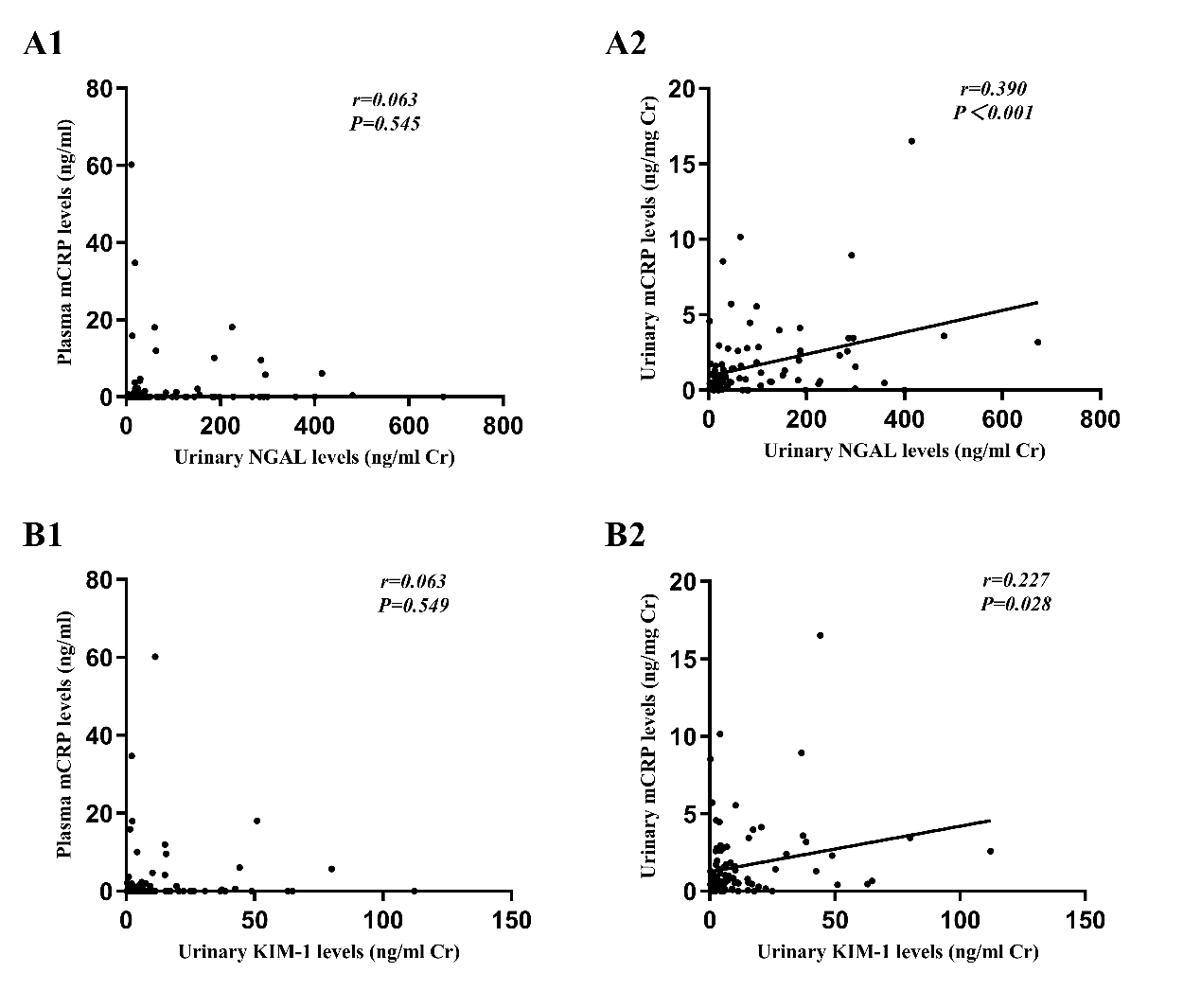


**Supplementary Figure S2:** Associations between plasma mCRP levels or urinary mCRP levels and tubulointerstitial injury features of lupus nephritis. (A1, B1) The association between plasma mCRP levels and urinary NGAL levels and KIM-1 levels in patients with lupus nephritis. (A2, B2) The associations between urinary mCRP levels and urinary NGAL levels and KIM-1 levels in patients with lupus nephritis. mCRP: modified C reactive protein; NGAL: neutrophil gelatinase-associated lipocalin; KIM-1: kidney injury molecule-1.


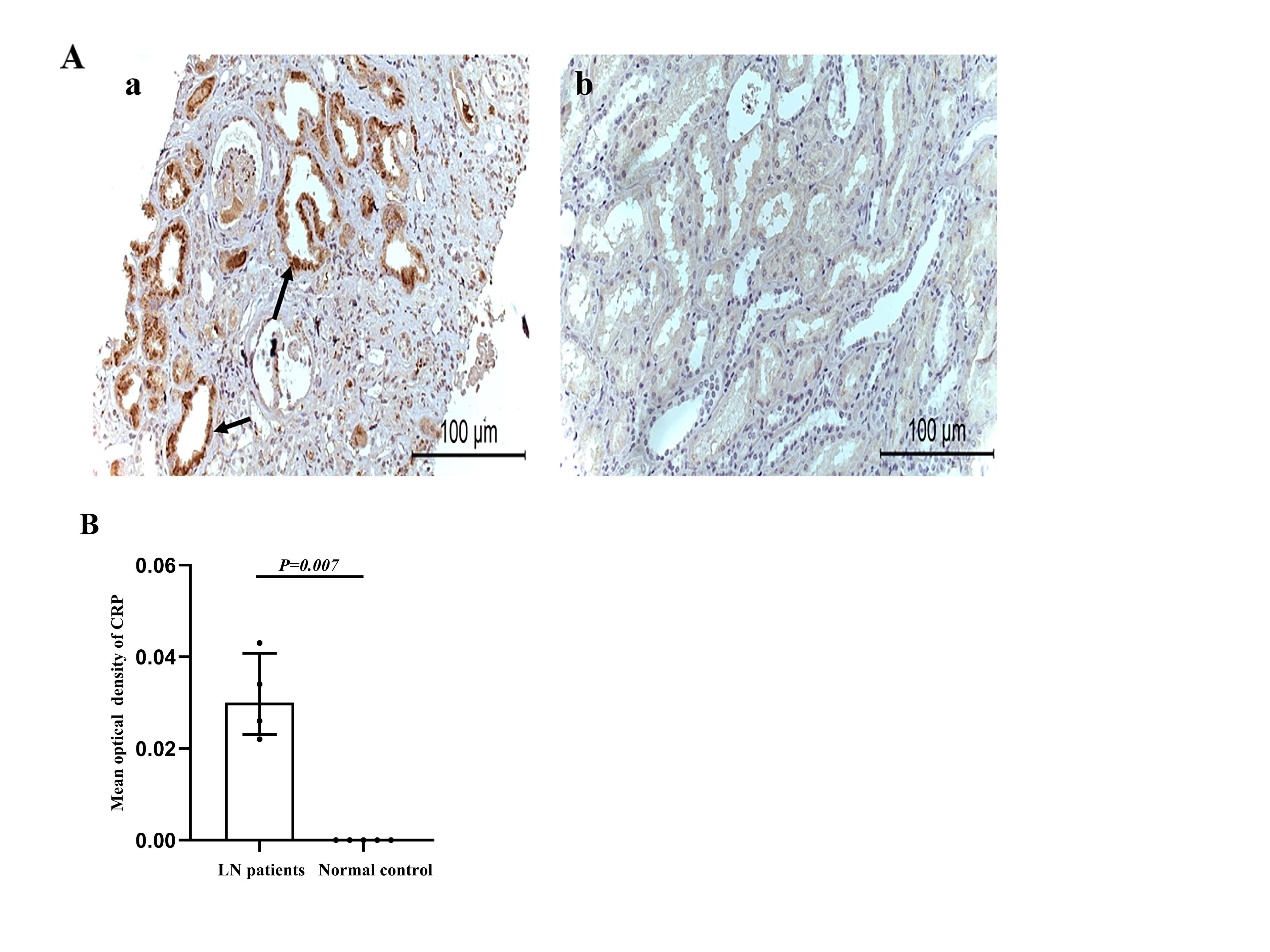


**Supplementary Figure S3:** Immunohistochemistry staining of CRP in renal biopsies of patients with autoimmune-related tubulointerstitial nephritis. (A-a) CRP staining was markedly positive in the tubules of patients with lupus-related tubulointerstitial nephritis (arrow). (A-b) CRP staining was barely seen in the tubules of normal kidneys (×400). Scale bars 100 μm. (B) The mean optical density of CRP in patients with lupus-related tubulointerstitial nephritis and normal controls. CRP: C reactive protein; LN: lupus nephritis.
